# Specific Recognition and Adsorption of Volatile Organic Compounds by Using MIL-125-Based Porous Fluorescence Probe Material

**DOI:** 10.3390/nano13192732

**Published:** 2023-10-09

**Authors:** Qiuyu Wu, Feiyang Tian, Wenqian Chen, Jianying Wang, Bo Lei

**Affiliations:** Key Laboratory of Organic Compound Pollution Control Engineering, Ministry of Education, School of Environmental and Chemical Engineering, Shanghai University, Shanghai 200444, China; wuqiuyuhua@126.com (Q.W.); feiyangtian3638@163.com (F.T.)

**Keywords:** metal organic frameworks, porous fluorescent probe, VOCs

## Abstract

The severity of the volatile organic compounds (VOCs) issue calls for effective detection and management of VOC materials. Metal-organic frameworks (MOFs) are organic-inorganic hybrid crystals with promising prospects in luminescent sensing for VOC detection and identification. However, MOFs have limitations, including weak response signals and poor sensitivity towards VOCs, limiting their application to specific types of VOC gases. To address the issue of limited recognition and single luminosity for specific VOCs, we have introduced fluorescent guest molecules into MOFs as reference emission centers to enhance sensitivity. This composite material combines the gas adsorption ability of MOFs to effectively adsorb VOCs. We utilized (MIL-125/NH_2_-MIL-125) as the parent material for adsorbing fluorescent molecules and selected suitable solid fluorescent probes (FGFL-B_1_) through fluorescence enhancement using thioflavin T and MIL-125. FGFL-B_1_ exhibited a heightened fluorescence response to various VOCs through charge transfer between fluorescent guest molecules and ligands. The fluorescence enhancement effect of FGFL-B_1_ on tetrahydrofuran (THF) was particularly pronounced, accompanied by a color change from yellow to yellowish green in the presence of CCl_4_. FGFL-B_1_ demonstrated excellent adsorption properties for THF and CCl_4_, with saturated adsorption capacities of 655.4 mg g^−1^ and 811.2 mg g^−1^, respectively. Furthermore, FGFL-B_1_ displayed strong luminescence stability and reusability, making it an excellent sensing candidate. This study addresses the limitations of MOFs in VOC detection, opening avenues for industrial and environmental applications.

## 1. Introduction

Environmental pollution has become a pressing global issue with the release of hazardous chemical pollutants into the atmosphere through industrial emissions, steam leakage, and fossil fuel combustion [1,2]. Among these pollutants, volatile organic compounds (VOCs) pose a significant threat to human health [3]. There is an urgent need to develop VOC detection sensors with high sensitivity capable of detecting a variety of gases. The selection of sensor materials plays a crucial role in achieving efficient VOC detection [4]. Metal-organic frameworks (MOFs) are highly effective porous materials for the removal of harmful gases [5,6,7]. They offer numerous advantages over traditional materials such as zeolite-type materials [8], mesoporous silica [9], resins [10], and activated carbons with additives [11,12]. Unlike zeolite-related inorganic hybrid materials that require a template for formation, MOFs rely primarily on a solvent as the main templating molecule [13,14]. To ensure efficient capture of harmful gases through interaction with MOFs, it is crucial to employ MOFs with appropriate pore size and shape [15]. MOFs are functional materials known for their tailorable porosity, making them attractive candidates for VOC capture and sensing [16].

MOFs are a class of crystalline micro-mesoporous hybrid materials with diverse potential applications [17,18,19,20]. They possess designable structures, adjustable chemical functions, a low-density skeleton, an ultra-high specific surface area, and functional permanent pore space [21,22,23]. Due to their porous structure and broad luminescence behavior, MOFs are commonly used as gas adsorbents and luminescent sensors [24,25,26,27]. These MOF sensors exhibit rapid response, high sensitivity, and non-invasive operation [28,29,30]. Luminescent MOF sensors often utilize lanthanide elements as metal centers or specific organic ligands [31,32,33,34]. The selection of materials for such sensors still has limitations [35]. The disadvantage of MOFs is that excitation and emission energy transfer primarily occur within a single component, making fluorescence advantageous only for specific volatile organic compounds (VOCs) that closely interact with the luminescent groups [35,36,37]. 

Introducing luminescent groups in MOFs as reference emission centers is a simple and effective strategy to enhance the luminescence sensitivity of MOF sensors, allowing for widespread applications. Yao et al. used a porous supramolecular framework to adsorb guest dye molecules, creating solid materials with high fluorescence. This provides a simple, low-cost, and efficient way to create luminescent solid materials [38]. Similarly, Tian created solid luminous materials by adsorbing guest dye molecules into a symmetrical urea-based polypore supramolecular assembly of tetramethyl calabinus [6] for the detection of VOCs [39]. By adopting this method, luminescent groups can be introduced into MOFs as reference emission centers, effectively addressing the issue of limited luminescence and specific VOC recognition in traditional MOFs. Furthermore, taking advantage of the excellent gas adsorption performance of MOFs, there is potential to utilize them as parent materials for simultaneous VOC detection and adsorption. MIL-125, a popular Ti-based MOF, stands out due to its high thermal stability, solid framework, large specific surface area, and two types of pore cages (effective reachable diameters of 12.55 Å and 6.13 Å, respectively, are octahedral and tetrahedral cages, connected by triangular windows of 5–7 Å). These properties make MIL-125 an exceptional material for gas adsorption [40]. In 2014, Kim et al. reported the adsorption and catalytic properties of MIL-125 and NH_2_-MIL-125 [41]. Recently, Kim et al. found that the formaldehyde adsorption ability of NH_2_-MIL-125 was significantly better than that of other MOF materials [42]. However, MIL-125 and NH_2_-MIL-125 have not been widely utilized for VOC detection. Our objective is to employ MIL-125 and NH_2_-MIL-125 as parent materials for developing fluorescent materials capable of detecting and adsorbing VOCs. 

The fluorescence probe FGFL-B_1_ (composed of MIL-125 and thioflavin T dye molecules) showed the strongest fluorescence enhancement effect on tetrahydrofuran (THF), and it exhibited distinct fluorescence discoloration when specifically recognizing CCl_4_. This method not only has a simple material fabrication step and strong operability but also expands the application range of MIL-125 and other MOF materials as fluorescent probes. The study aimed to address practical challenges in VOC detection and treatment, offering potential applications in industrial and living environments.

## 2. Experimental

### 2.1. Materials

All reagent-grade chemicals were used without further purification: NH_2_-terephthalate acid (H_2_ATA, RG, Adamas-beta, Titan, Shanghai, China), terephthalate acid (H_2_BDC, RG, Adamas-beta, Titan, Shanghai, China), tetrabutyl titanate (TBT, AR, Greagent, Titan, Shanghai, China), anhydrous methanol (AR, Greagent, Titan, Shanghai, China), N, N-dimethylformamide (DMF, AR, Greagent, Titan, Shanghai, China), dansyl chloride (Leyan, Shanghai, China), pyren-1-amine (bidepharm, Shanghai, China), 8-hydroxyquinoline (Adamas-beta, Titan, Shanghai, China), umbelliferone (Adamas-beta, Titan, Shanghai, China), thioflavin T (ThT, bidepharm, Shanghai, China) benzene (C_6_H_6_, AR, General-Reagent, Titan, Shanghai, China), toluene (C_6_H_5_CH_3_, AR, General-Reagent, Titan, Shanghai, China), dichloromethane (CH_2_Cl_2_, AR, General-Reagent, Titan, Shanghai, China), carbon tetrachloride (CCl_4_, AR, General-Reagent, Titan, Shanghai, China), formaldehyde (HCHO, AR, General-Reagent, Titan, Shanghai, China), and tetrahydrofuran (C_4_H_8_O, THF, Safe Dry, Adamas-beta, Titan, Shanghai, China).

### 2.2. Methods

#### 2.2.1. Precursor Synthesis

The MIL-125 and NH_2_-MIL-125 were prepared by a solvothermal method, which was modified from a previous report [43].

Terephthalate acid (H_2_BDC, 3 mmol) was completely dissolved in N, N-dimethylformamide (DMF, 9 mL), and anhydrous methanol (3 mL). Then, 0.75 mmol of tetrabutyl titanate (TBT) was added to the solution under an N_2_ atmosphere to form a suspension. After vigorous stirring for 30 min, the suspension was reacted in a 50 mL Teflon-sealed autoclave at 423 K for 24 h. Finally, the material was cooled down to room temperature. The white MIL-125 powders were obtained after being washed with DMF and anhydrous methanol, dried at 333 K, and then vacuum dried at 423 K for 3 h (In order to remove water and DMF). Yellow NH_2_-MIL-125 powders were prepared in the same way, except that H_2_BDC was replaced with NH_2_-terephthalate acid (H_2_ATA).

#### 2.2.2. Porous Fluorescence Probe Synthesis

15 mg of each of the five dyes (thioflavin T (ThT), 8-hydroxyquinoline, dansyl chloride, pyren-1-amine, umbelliferone) were placed in five small bottles containing 20 mL acetonitrile. Ultrasonic treatment was carried out for 15 min to form a uniformly dispersed dye solution. Then 50 mg of NH_2_-MIL-125/MIL-125 was added to this uniformly dispersed dye solution and let stand for 3.5 h. After centrifugation, the lower layer was precipitated and dried at 333 K for 12 h to obtain (FGFL-A_1–5_ and FGFL-B_1–5_) materials. In this study, MIL-125/NH_2_-MIL-125 was used as the parent material to adsorb five fluorescent dye molecules (thioflavin T, 8-hydroxyquinoline, dansyl chloride, pyrene-1 amine, and cymflorone), labeled as 1, 2, 3, 4, and 5 in the paper. Different composites were prepared, with the complex formed by MIL-125 named FGFL-B_1-5_ and the composites formed by NH_2_-MIL-125 named FGFL-A_1–5_. By calculating the mass of dye before and after adsorption, the amounts of fluorophores 1–5 in 50 mg MIL-125 were 6.7 mg, 4.8 mg, 1.4 mg, 1.1 mg, and 5.4 mg, respectively, and 5.3 mg, 2.9 mg, 1 mg, 0.9 mg, and 2.3 mg in 50 mg of NH_2_-MIL-125, respectively.

### 2.3. Adsorption and Fluorescence Response of FGFL-A_1–5_/FGFL-B_1–5_ for VOCs

A glass bottle (10 mL) containing 50 mg of FGFL-A_1–5_/FGFL-B_1–5_ was placed into the sealable glass container (100 mL) and vacuumed with a vacuum pump until the weight of the FGFL-A_1–5_/FGFL-B_1–5_ remained the same. Then, another glass bottle (10 mL) containing a small amount of volatile organic compounds (C_6_H_6_, C_6_H_5_CH_3_, THF, CCl_4_, C_2_Cl_2_, HCHO) was placed in the vacuum-sealed glass container and sealed for 3.5 h. After the adsorption of different VOCs, the fluorescence of FGFL-A_1–5_/FGFL-B_1–5_ was detected successively. The weight variation was measured, and corresponding solid-state fluorescence spectra were determined at intervals of approximately 20–240 min over several hours in order to obtain the vapor adsorption profile.

### 2.4. Measurement of Fluorescence Spectra of Solid FGFL-A_1–5_/FGFL-B_1–5_

Fluorescence spectra of solid FGFL-A_1–5_/FGFL-B_1–5_ before and after adsorbing VOCs were recorded at room temperature by using a fluorescence spectrometer (F-320 PL, Guangdong Technology, Guangzhou, China), respectively.

### 2.5. Characterization

A powder X-ray diffractometer (PXRD, Bruker D8 Advance) was used to characterize the crystal structure of the synthesized catalysts by Cu Kα radiation operated at 40 kV and 40 mA. Patterns were collected using a scan speed of 2 s/step and a step size of 0.02° at a 2θ range from 5° to 50°. The morphologies and microstructures of the prepared materials were observed using a scanning electron microscope (SEM, Gemini SEM300, ZEISS, Germany). The Brunauer–Emmett–Teller (BET) surface areas of the samples were measured using a nitrogen adsorption instrument (Micromeritics ASAP 2460 analyzer, Beishide, Beijing, China) at liquid-nitrogen temperature. The IR experiments were carried out on a Nicolet 380 FT-IR spectrometer. The IR spectra of pure samples were collected without diluting with KBr. Photoluminescence (PL) spectra were recorded using a Guangdong Technology F-320 PL spectrophotometer with an excitation wavelength of 385 nm. All the liquid-state ^1^H NMR spectra in this work were obtained on a Bruker 400 MHz spectrometer in dimethyl sulfoxide solution. Ultraviolet-visible (UV-vis) diffuse reflectance spectra (DRS) of the prepared samples were recorded using a UV-vis spectrometer (UV-2600, Shimadzu, Kyoto, Japan) with a background of BaSO_4_.

## 3. Results and Discussion

### 3.1. Structure and Property of FGFL-A_1–5_/FGFL-B_1–5_

**Figure 1:**
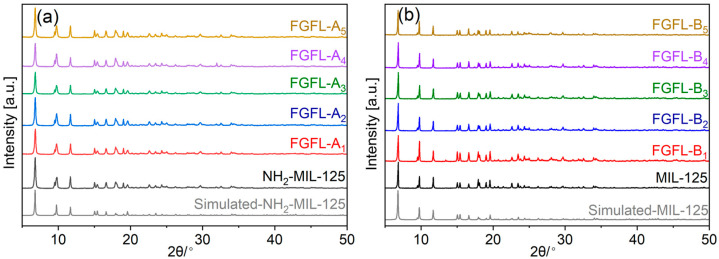
PXRD patterns of (**a**) NH_2_-MIL-125, FGFL-A_1–5_, and (**b**) MIL-125, FGFL-B_1–5_ (Thioflavin T, 8-hydroxyquinoline, dansyl chloride, pyrene-1 amine, and umbelliferone were referred to as 1, 2, 3, 4 and 5).

We successfully synthesized MIL-125 and NH_2_-MIL-125 by the solvothermal method [40]. The powder X-ray diffraction (PXRD) patterns are shown in Figure 1. The main diffraction peaks of NH_2_-MIL-125 (Figure 1a) and MIL-125 (Figure 1b) were consistent with the simulated spectra, indicating that both MIL-125 and NH_2_-MIL-125 have been successfully prepared [40]. Different fluorescent probe materials were prepared by introducing dye molecules into MIL-125/NH_2_-MIL-125 by the impregnation method. The PXRD pattern of NH_2_-MIL-125/MIL-125 (FGFL-A_1–5_/FGFL-B_1–5_) remained unchanged after the adsorption of fluorescent dye molecules, indicating that the crystal structure of NH_2_-MIL-125/MIL-125 was not affected by the adsorption process.

**Figure 2:**
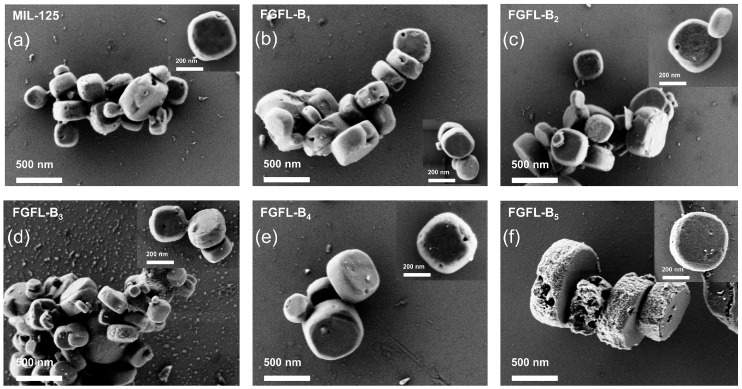
(**a**–**f**) SEM images of MIL-125, FGFL-B_1_, FGFL-B_2_, FGFL-B_3_, FGFL-B_4,_ and FGFL-B_5_, respectively.

To investigate the impact of dye molecules on the morphology of NH_2_-MIL-125/MIL-125, we examined both the pristine materials and composites using a Scanning Electron Microscope (SEM). Figure 2 shows that MIL-125 and FGFL-B_1–5_ exhibit similar disc-like shapes with a diameter of approximately 300 nm and a thickness of around 100 nm. Similarly, the morphology and size of NH_2_-MIL-125 and FGFL-A_1–5_, as shown in Figure S1, remained unchanged. The surface morphology and structure, as observed from the PXRD and SEM patterns, also did not show significant alterations, and no dye was detected.

**Figure 3:**
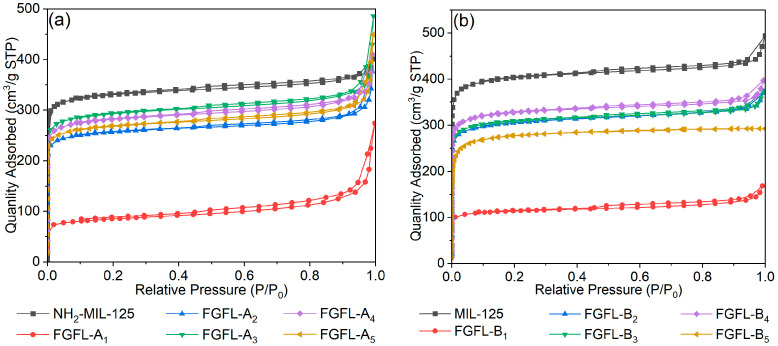
N_2_ adsorption–desorption isotherms of (**a**) NH_2_-MIL-125, FGFL-A_1–5_, (**b**) MIL-125, FGFL-B_1–5_.

Nitrogen sorption experiments demonstrated the highly porous nature of NH_2_-MIL-125 and MIL-125, characterized by a type I isotherm typical of micro-porous solids (Figure 3), which exhibited a BET surface area of 1031.60 m^2^ g^™1^ and 1252.45 m^2^ g^−1^, and a micro-pore volume of 0.4806 cm^3^ g^−1^ and 0.6015 cm^3^ g^−1^, respectively (Figure S2, Table S1). Compared to NH_2_-MIL-125/MIL-125, the specific BET surface area and micro-pore volume of FGFL-A_1–5_/FGFL-B_1–5_ were significantly reduced, indicating successful adsorption of fluorescent dye molecules onto NH_2_-MIL-125/MIL-125 (Table S1). Notably, the thioflavin T (ThT) composite exhibited the minimum BET surface area and micro-pore volume. In the case of FGFL-A_1_, formed using NH_2_-MIL-125 as a template, the BET surface area decreased from 1031.60 m^2^ g^−1^ to 271.45 m^2^ g^−1^, and the micropore volume decreased from 0.4806 cm^3^ g^−1^ to 0.1000 cm^3^ g^−1^. The BET surface area of MIL-125 composite (FGFL-B_1_) decreased from 1252.25 m^2^ g^−1^ to 356.05 m^2^ g^−1^, and the micro-pore volume decreased from 0.6015 cm^3^ g^−1^ to 0.1615 cm^3^ g^−1^. Therefore, NH_2_-MIL-125/MIL-125 exhibited the highest ThT adsorption capacity.

### 3.2. Fluorescence of FGFL-A_1–5_/FGFL-B_1–5_

**Figure 4:**
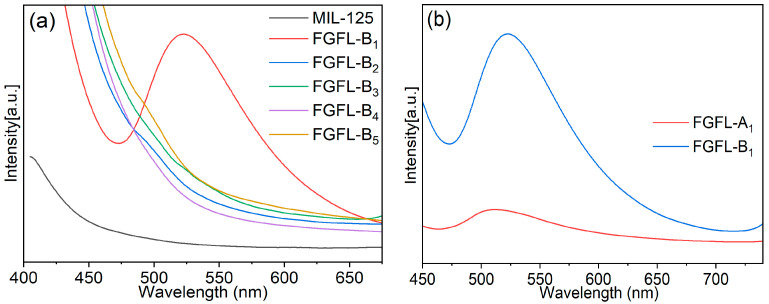
(**a**) Fluorescence spectra of MIL-125 and FGFL-B_1–5_, (**b**) Fluorescence spectra of FGFL-A_1_ and FGFL-B_1_.

The fluorescence spectra of FGFL-A_1–5_/FGFL-B_1–5_ were tested to identify the optimal fluorescent probe materials. The results revealed that ThT exhibited a strong fluorescence enhancement effect when adsorbed onto NH_2_-MIL-125/MIL-125 (Figure 4, Figure S3 and Figure S10), while other dye molecules showed minimal fluorescence response. Therefore, ThT was deemed the most suitable fluorescent guest molecule for NH_2_-MIL-125/MIL-125. Additionally, FGFL-B_1_ demonstrated significantly better fluorescence enhancement than FGFL-A_1_ (Figure 4b). The electrophotographs captured under sunlight and UV (365 nm) illumination (Figure S4) demonstrated that FGFL-B_1_, a fluorescent material formed after ThT adsorption by MIL-125, emitted vibrant yellow light under UV (365 nm) illumination.

**Figure 5:**
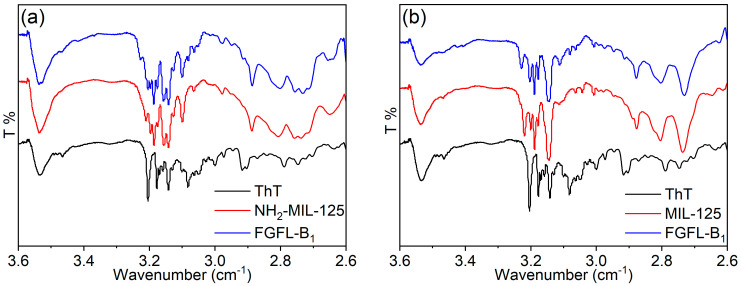
(**a**) IR spectra of ThT, NH_2_-MIL-125, and FGFL-A_1_, (**b**) IR spectra of ThT, MIL-125, and FGFL-B_1_.

To analyze whether ThT undergoes simple physical adsorption or chemical coupling with MIL-125/NH_2_-MIL-125, we conducted IR spectroscopy on ThT, NH_2_-MIL-125/MIL-125, and FGFL-A_1–5_/FGFL-B_1–5_. As shown in Figure 5, the carboxyl group characteristic peak of NH_2_-MIL-125/MIL-125 showed at 3.140–3.204 (log1380–log1600 cm^−1^) [40]. An evident larger wavenumber shift at 3.23 (log1700 cm^−1^) was observed in the infrared spectra of FGFL-A_1–5_/FGFL-B_1–5_, indicating the adsorption and coupling of ThT molecules with NH_2_-MIL-125/MIL-125. The larger wavenumber shift was more pronounced in the FGFL-B_1_ infrared spectra. Figure S5 displays the Raman spectra of NH_2_-MIL-125/MIL-125, ThT, and FGFL-A_1_/FGFL-B_1_, with FGFL-A_1_/FGFL-B_1_ revealing peaks from both NH_2_-MIL-125/MIL-125 and the fluorescent dye ThT. Additionally, Figure S6a,b illustrates the liquid nuclear magnetic hydrogen spectra of ThT, MIL-125, and FGFL-B_1_. The peak at 8.14 ppm of ThT belongs to the H peak on the benzene ring on the benzylamine ring; the H peak (8.06 ppm) on the benzene ring of the organic ligand terephthalic acid of MIL-125 was significantly offset in FGFL-B_1_, indicating that ThT forms H···O conjugations with MIL-125 [44]. The ThT molecule consists of a benzylamine ring and a phenyl sulfide ring. When the benzylamine ring and phenyl sulfide ring rotate freely around the C-C bond in the natural state, the ThT fluorescence signal is weak. Once this rotation is limited by some structures, the ThT fluorescence is enhanced [45,46,47]. When ThT fluorescent guest molecules were introduced into NH_2_-MIL-125/MIL-125 as light sources and coupled with them via H···O interaction, the restriction of intramolecular rotation (RIR) of ThT fluorescence was caused to some extent. Charge transfer from fluorescent guest molecules to organic ligands was formed, resulting in a strong fluorescence response [48,49,50]. In order to confirm this phenomenon, we tested the solid UV-vis absorption spectra of ThT, MIL-125, and FGFL-B_1_, respectively. As can be seen from Figure S7a, the absorption peak of FGFL-B_1_ formed by the combination of ThT and MIL-125 had a significant red shift compared with MIL-125. As a chromophore, ThT is prone to electron transition. The FGFL-B_1_ material formed by the combination of MIL-125 and ThT had a significant red shift relative to the absorption wavelength of MIL-125 and a significant blue shift relative to the absorption wavelength of ThT, which corresponded to the above phenomenon [51,52]. The band gaps of ThT, MIL-125, and FGFL-B_1_ can be calculated from the solid UV-vis absorption spectra using the Kubelka–Munk function [53]. It can be seen from Figure S7b that by combining with ThT, the band gap of MIL-125 was reduced from 3.4 eV to 2.4 eV.

### 3.3. Fluorescence Sensors and Storage Performance of VOCs over FGFL-B_1_

Environmental pollution is a pressing global issue caused by the discharge of harmful chemical pollutants into the atmosphere. Volatile organic compounds (benzene, toluene, tetrahydrofuran, carbon tetrachloride, dichloromethane, methylene chloride, formaldehyde) are the main cause of environmental air pollution and pose risks to human health [54,55,56]. For instance, THF exhibits stimulatory and anesthetic effects. Inhalation causes upper respiratory irritation, nausea, dizziness, headache, and central nervous system depression [57,58]. CCl_4_ and its decomposition products can be absorbed through the respiratory tract, and skin contact can result in rapid absorption. CCl_4_ is particularly damaging to peripheral nerves, especially the liver [59]. Therefore, the development of fluorescent probes and porous materials for the detection and adsorption of VOC gases is crucial to addressing this issue.

**Figure 6:**
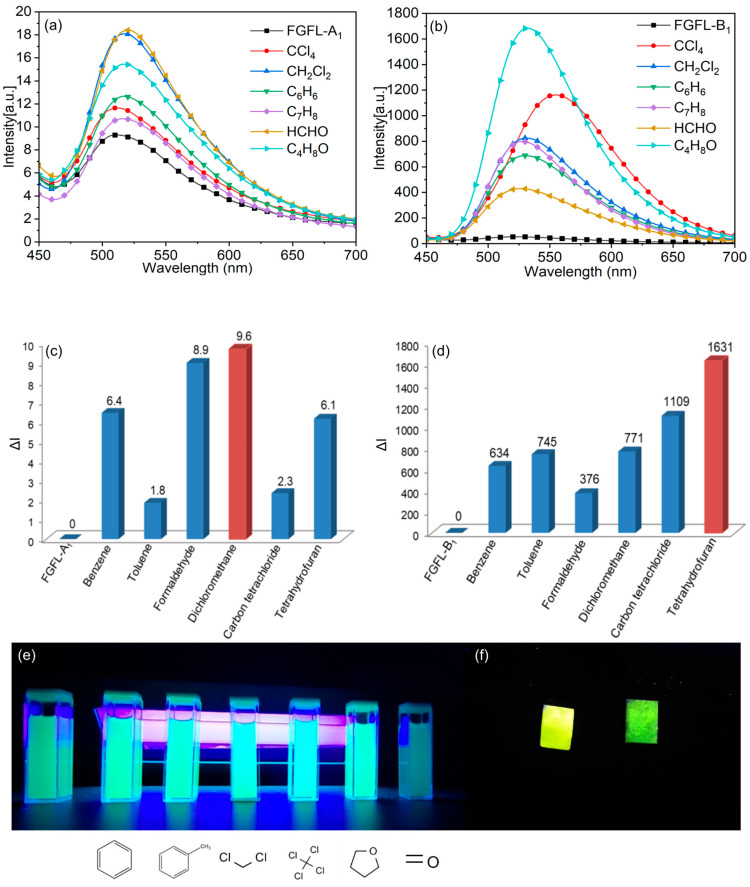
A general survey of the fluorescence spectra of (**a**) FGFL-A_1_ and (**b**) FGFL-B_1_ loaded with the six selected VOCs. Relative fluorescence intensities of (**c**) FGFL-A_1_ and (**d**) FGFL-B_1_ in response to the six selected VOCs. (**e**) Photos under UV (365 nm) of FGFL-B_1_ and FGFL-B_1_ loaded with six selected VOCs. (**f**) Photos under UV (365 nm) of FGFL-B_1_ (**left**) and FGFL-B_1_ loaded with CCl_4_ (**right**).

In this paper, fluorescent molecules were selected through fluorescence spectra testing to determine the most suitable candidates. FGFL-A_1_ and FGFL-B_1_ were chosen as fluorescent probes for investigating the detection and adsorption properties of six common volatile organic compounds (VOCs): dichloromethane (CH_2_Cl_2_), carbon tetrachloride (CCl_4_), tetrahydrofuran (THF), formaldehyde (HCHO), benzene (C_6_H_6_), and toluene (C_6_H_5_CH_3_). As shown in Figure 6a,b, FGFL-B_1,_ and FGFL-A_1_ demonstrated varying degrees of fluorescence response towards the VOCs. However, the fluorescence enhancement effect of FGFL-A_1_ was very low, which was negligible compared with that of FGFL-B_1_ (Figure 6c,d). Therefore, FGFL-B_1_ is considered the most suitable fluorescent probe material. After FGFL-B_1_ and FGFL-B_1_ adsorbed with different VOCs were formed into a suspension in acetonitrile solvent, different fluorescence responses could be clearly observed under UV light (Figure 6e). Notably, FGFL-B_1_ showed the most significant fluorescence enhancement effect towards THF gas, with fluorescence intensity 36 times higher than FGFL-B_1_ (Figure 6b). Additionally, the adsorption of CCl_4_ gas induced a noticeable red shift in FGFL-B_1_ (Figure 6b). This red shift resulted in a distinct transformation in fluorescence color from yellow to yellowish green (After the adsorption of CCl_4_, the maximum emission peak of FGFL-B_1_ undergoes a red shift from 530 nm to 560 nm, while it remained unchanged at 530 nm after the adsorption of other VOCs). To demonstrate the color transformation more effectively, test strips of FGFL-B_1_ were fabricated, which visibly turned yellowish green upon adsorption of CCl_4_ (Figure 6f). This test strip method offers the potential for faster and easier detection of simulated factory or indoor gas leaks.

**Figure 7:**
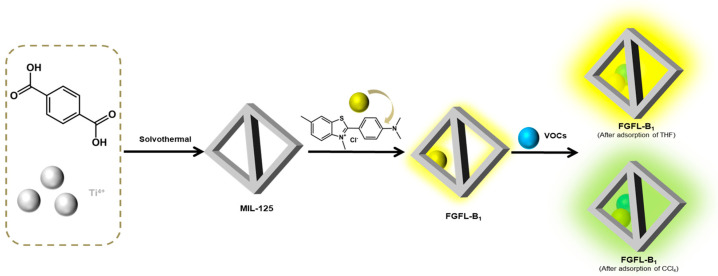
Schematic diagram of FGFL-B_1_ adsorption detection of VOCs.

FGFL-B_1_ was formed by the recombination of ThT (4 Å) within the MIL-125 channel [60,61]. Upon the entry of VOC gas into the FGFL-B_1_ channel, collisions with ThT molecules generate an effect [62]. The interaction between ThT and MIL-125 restricts the rotation of the benzene ring, thereby limiting non-radiative energy consumption pathways and reinforcing radiative transitions. As a result, fluorescence is substantially enhanced [63,64,65]. To illustrate this effect, we present the example of THF adsorption (Figure S8). Following the adsorption of THF, the fluorescence intensity of FGFL-B_1_ was significantly enhanced, accompanied by a change in the full width at half maximum (FWHM) from 73.63 nm to 85.04 nm [66]. Electronegative molecules induce a pronounced red shift in the spectra of the material. CCl_4_, known for its strong electronegativity, triggers intramolecular charge transfer of ThT, resulting in a red shift effect and fluorescence color transformation [67,68]. Therefore, FGFL-B_1_ can selectively recognize CCl_4_. These findings demonstrate that the incorporation of fluorescent guest molecules (ThT) as luminescent clusters significantly improves the fluorescence detection performance of materials and overcomes the limitations of conventional MOFs, which only exhibit a fluorescence response to specific gases (Figure 7).

**Figure 8:**
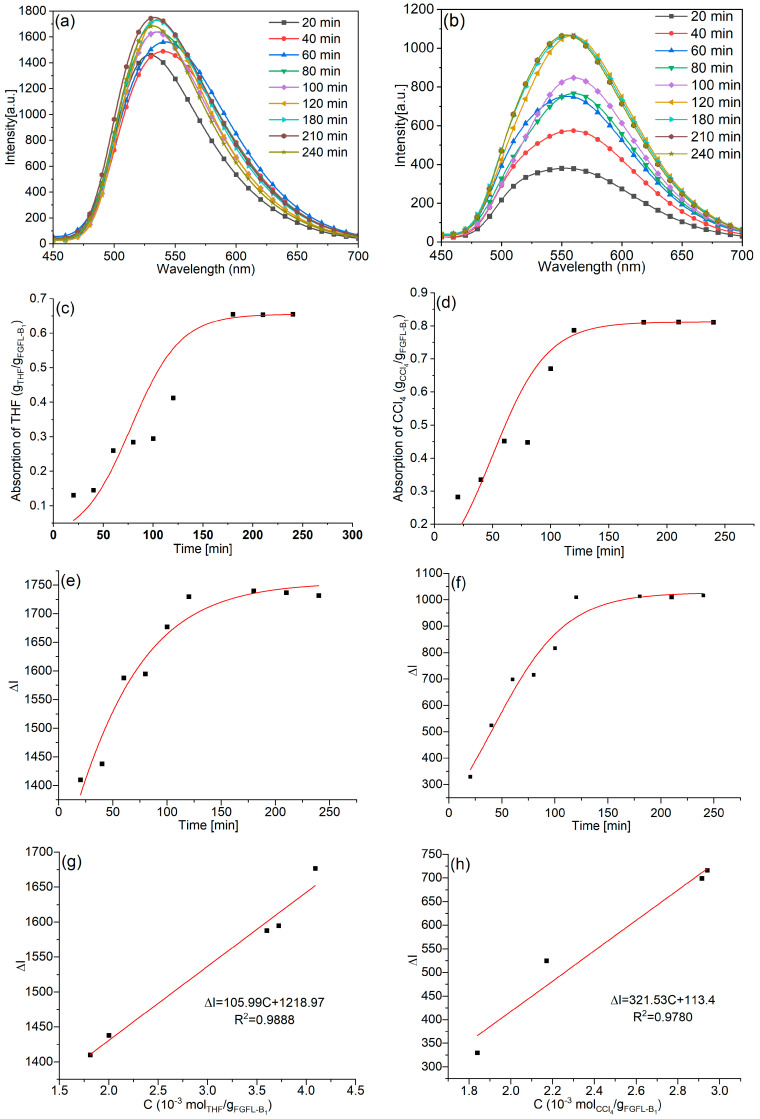
The titrated fluorescence spectra of FGFL-B_1_ loaded (**a**) THF and (**b**) CCl_4_. Adsorption profile of the loading of (**c**) THF and (**d**) CCl_4_ in FGFL-B_1_ upon increasing the adsorption time. Change in the fluorescence intensity of FGFL-B_1_ by loading (**e**) THF and (**f**) CCl_4_ upon increasing the adsorption time. Plot of ΔI versus the amount of (**g**) THF and (**h**) CCl_4_ adsorbed by solid FGFL-B_1_**.**

The titrated fluorescence spectra of FGFL-B_1_ were analyzed in detail to monitor the real-time adsorption of THF and CCl_4_ (Figure 8a,b). The fluorescence intensity of solid FGFL-B_1_ at 530 nm and 560 nm increased with the increase in the adsorption time of THF and CCl_4_. As shown in Figure 8e,f, it took approximately 2 h for FGFL-B_1_ to reach its maximum fluorescence intensity with THF and CCl_4_, while only about 20 min were needed to shift the peak position to 560 nm. Although the fluorescence intensity and peak position of FGFL-B_1_ remained unchanged, the absorption rates of THF and CCl_4_ continued to increase within 3 h (Figure 8c,d). Comparing Figure 8c–f, it can be observed that the adsorption amount and time of THF and CCl_4_ required to achieve the maximum equilibrium value of FGFL-B_1_ fluorescence intensity are much lower than those required by saturated adsorption. The size of ThT (4 Å) is large, and the barrier entering porous MIL-125 is much larger than that of VOCs molecules. Therefore, the amount of ThT that can interact with MIL-125 is significantly lower than that of VOC molecules. Therefore, the time required for fluorescence to reach maximum equilibrium and the amount of gas is far less than the time of saturation adsorption and gas volume. By comparing the mass of FGFL-B_1_ before and after the saturated adsorption of THF and CCl_4_, we calculated that the adsorption properties of FGFL-B_1_ for THF and CCl_4_ were 655 mg g^−1^ and 812 mg g^−1^, respectively (Table S2). The adsorption performance of other VOC gases is shown in Table S1. Additionally, the ΔI curves representing the adsorption capacity of THF and CCl_4_ on FGFL-B_1_ (Figure 8g,h) were plotted using the adsorption-time curve data (Figure 8c,d) and intensity (ΔI) versus time curve data (Figure 8e,f) obtained from the interpolation method. The detection limits (DL) [39] of FGFL-B_1_ for THF and CCl_4_ were determined to be 1.41 × 10^−4^ mol g^−1^ and 4.66 × 10^−5^ mol g^−1^, respectively.

The detection of VOCs by FGFL-B_1_ is based on a fluorescence reaction triggered by the interaction of gases after entering the material. VOCs can be detached from FGFL-B_1_, allowing the material to be recycled [39]. To validate this, we conducted adsorption and desorption experiments using THF and CCl_4_ as examples. Lifetime tests demonstrated that the adsorption and desorption of THF and CCl_4_ on FGFL-B_1_ were not only reversible but also highly stable. Numerous adsorption and desorption experiments consistently indicated that the detection properties of FGFL-B_1_ for THF and CCl_4_, such as fluorescence intensity, remained unchanged (Figure S9c,d). Furthermore, a comparison of the PXRD patterns of FGFL-B_1_ before and after the adsorption and desorption of THF and CCl_4_ revealed no significant changes, suggesting that the adsorption or desorption of THF and CCl_4_ had minimal impact on FGFL-B_1_ (Figure S9a,b). These results provided strong evidence for the excellent stability of FGFL-B_1_.

## 4. Conclusions

We investigated the fluorescence enhancement effect of FGFL-B_1_, a composite of the ThT dye molecules and MIL-125 H···O conjugation, on VOCs by introducing various fluorescent dye molecules into MIL-125 and NH_2_-MIL-125 synthesized via the solvothermal method. The presence of steric hindrance in ThT molecules both hampers and enhances their intramolecular rotation, resulting in a remarkable fluorescence enhancement effect on THF. The fluorescence intensity increases by a factor of 36 upon THF adsorption. Moreover, due to the strong electronegativity of CCl_4_, FGFL-B_1_ exhibits selective recognition of CCl_4_, leading to a distinct yellow-to-yellowish-green fluorescence color change. Remarkably, FGFL-B_1_ demonstrates excellent adsorption capacities for THF and CCl_4_, with values of 655.4 mg g^−1^ and 811.2 mg g^−1^, respectively. As a result, the prepared porous fluorescence probe material, FGFL-B_1_, holds great potential for detecting and adsorbing mixed gases in both industrial and domestic environments. This method opens up new possibilities for fluorescence detection of adsorption properties in Ti-based MOF materials and other MOF materials. 

## Data Availability

The data presented in this study are available on request from the corresponding author.

## Supplementary Materials

The following supporting information can be downloaded at: https://www.mdpi.com/article/10.3390/nano13192732/s1, Figure S1. (a–f) SEM images of NH_2_-MIL-125, FGFL-A_1_, FGFL-A_2_, FGFL-A_3_, FGFL-A_4_ and FGFL-A_5_, respectively; Figure S2. The corresponding BJH pore size distribution of the as-prepared samples of (a) NH_2_-MIL-125, FGFL-A_1_, FGFL-A_2_, FGFL-A_3_, FGFL-A_4_ and FGFL-A_5_ (b) MIL-125, FGFL-B_1_, FGFL-B_2_, FGFL-B_3_, FGFL-B_4_ and FGFL-B_5_. Table S1: BET surface area and T-Plot micro-pore volume of NH_2_-MIL-125, FGFL-A_1–5_, MIL-125, and FGFL-B_1–5_; Figure S3. Fluorescence spectra of (a) FGFL-A_1_, (b) FGFL-A_2_, (c) FGFL-A_3_, (d) FGFL-A_4_ and (e) FGFL-A_5_; Figure S4. Electronic photos of ThT, MIL-125, and FGFL-B_1_ (a–c) under sunlight and (d–f) under UV (365 nm); Figure S5. Raman spectra of (a) ThT, NH_2_-MIL-125, and FGFL-A_1_, (b) ThT, MIL-125, and FGFL-B_1_; Figure S6. (a) The liquid-state ^1^H NMR spectra in dimethyl sulfoxide of ThT, FGFL-B_1,_ and MIL-125; (b) The liquid-state ^1^H NMR spectra in dimethyl sulfoxide of ThT, FGFL-B_1_, MIL-125 (7.87–8.25 ppm); (c) The solid-state ^1^H NMR spectra of MIL-125, FGFL-B_1_ and ThT; Figure S7. (a) The UV-vis absorption spectra of ThT, MIL-125, and FGFL-B_1_; (b) The plot of (ahv)^1/2^ versus hv of ThT, MIL-125, and FGFL-B_1_; Figure S8. The fluorescence spectra of FGFL-B_1_ and FGFL-B_1_ loaded with THF. Table S2: Adsorption properties of FGFL-B_1_ for the six selected VOCs; Figure S9. After FGFL-B_1_ adsorbed VOCs, the VOCs were removed by vacuum drying at 333 K. PXRD patterns and fluorescence intensity were measured before and after the cycle stability experiment. The numbers 1, 2, 3, 4, 5, and 6 represent the number of adsorption and desorption cycles. A general survey of the PXRD patterns of (a) FGFL-B_1_ before and after loading with CCl_4_, and (b) FGFL-B_1_ before and after loading with THF. A general survey of the fluorescence spectra of (c) FGFL-B_1_ before and after loading with CCl_4_, (d) FGFL-B_1_ before and after loading with THF; Figure S10. Fluorescence spectra of (a) FGFL-B_1_, (b) FGFL-B_2_, (c) FGFL-B_3_, (d) FGFL-B_4_, and (e) FGFL-B_5_.
