# Supplementary material for: Specific Recognition and Adsorption of Volatile Organic Compounds by Using MIL-125-Based Porous Fluorescence Probe Material
Source: Nanomaterials (Basel). 2023 Oct 9;13(19):2732. doi: 10.3390/nano13192732 (PMC10574030; doi:10.3390/nano13192732)
Supplement: Supplementary file 1 [file nanomaterials-13-02732-s001.zip › nanomaterials-2488674-supplementary.pdf]

Supplementary Materials

Specific Recognition and Adsorption of Volatile Organic Compounds by Using MIL-125-Based Porous Fluorescence Probe Material

Qiuyu Wu †, Feiyang Tian †, Wenqian Chen *, Jianying Wang * and Bo Lei *

Key Laboratory of Organic Compound Pollution Control Engineering, Ministry of Education, School of Environmental and Chemical Engineering, Shanghai University, Shanghai 200444, China; wuqiuyuhua@126.com (Q.W.); feiyangtian3638@163.com (F.T.)

* Correspondence: wenqianchen@shu.edu.cn (W.C.); wjytech@163.com (J.W.); lb870213@shu.edu.cn (B.L.)

† These authors contributed equally to this work.

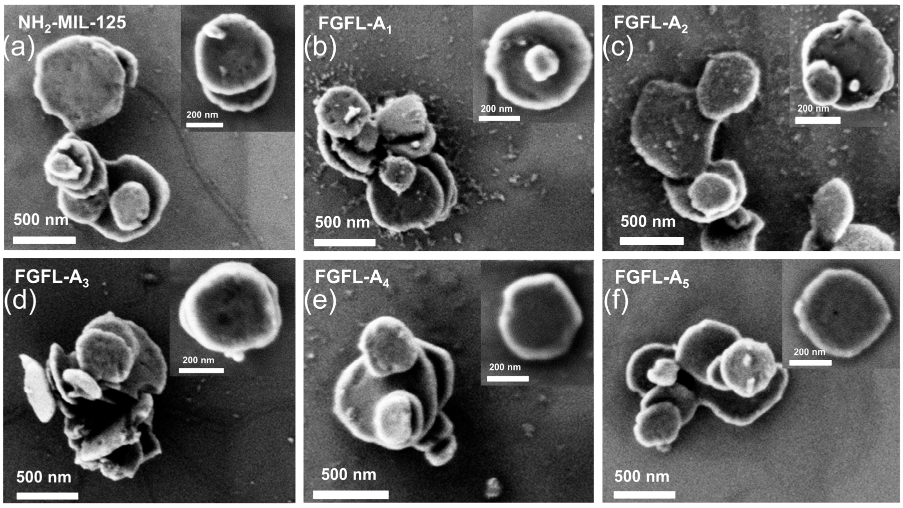

Figure S1. (a-f) SEM images of NH_2-MIL-125, FGFL-A_1, FGFL-A_2, FGFL-A_3, FGFL-A_4 and FGFL-A_5, respectively.

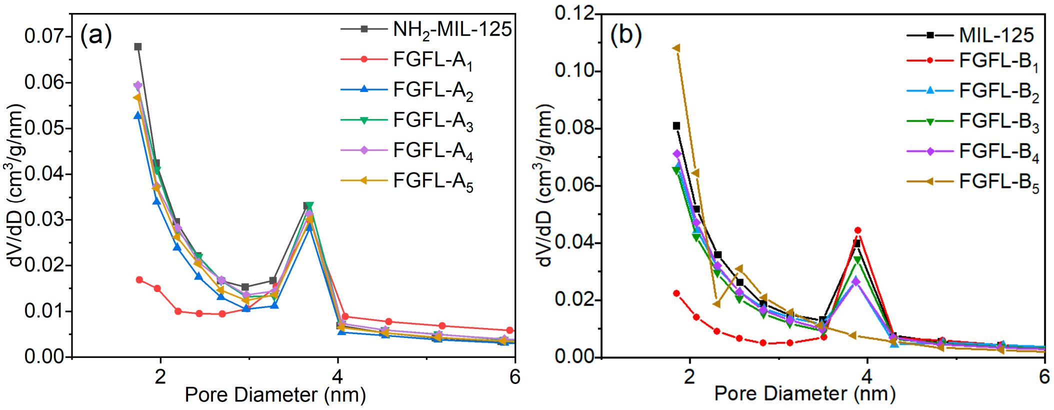

Figure S2. The corresponding BJH pore size distribution of the as-prepared samples of (a) NH_2-MIL-125, FGFL-A_1, FGFL-A_2, FGFL-A_3, FGFL-A_4 and FGFL-A_5 (b) MIL-125, FGFL-B_1, FGFL-B_2, FGFL-B_3, FGFL-B_4 and FGFL-B_5.

Table S1. BET surface area and T-Plot micro-pore volume of NH_2-MIL-125, FGFL-$A_{1\text{-}5}$, MIL-125 and FGFL-$B_{1\text{-}5}$.

Samples	BET surface area / m^2 g^{-1}	micro-pore volume / cm^3 g^{-1}
NH_2-MIL-125	1031.60	0.4806
FGFL-A_1	271.45	0.1000
FGFL-A_2	800.10	0.3766
FGFL-A_3	913.18	0.4267
FGFL-A_4	879.49	0.4062
FGFL-A_5	836.65	0.3903
MIL-125	1252.25	0.6015
FGFL-B_1	356.05	0.1615
FGFL-B_2	949.38	0.4410
FGFL-B_3	960.91	0.4597
FGFL-B_4	1018.07	0.4778
FGFL-B_5	862.28	0.4119

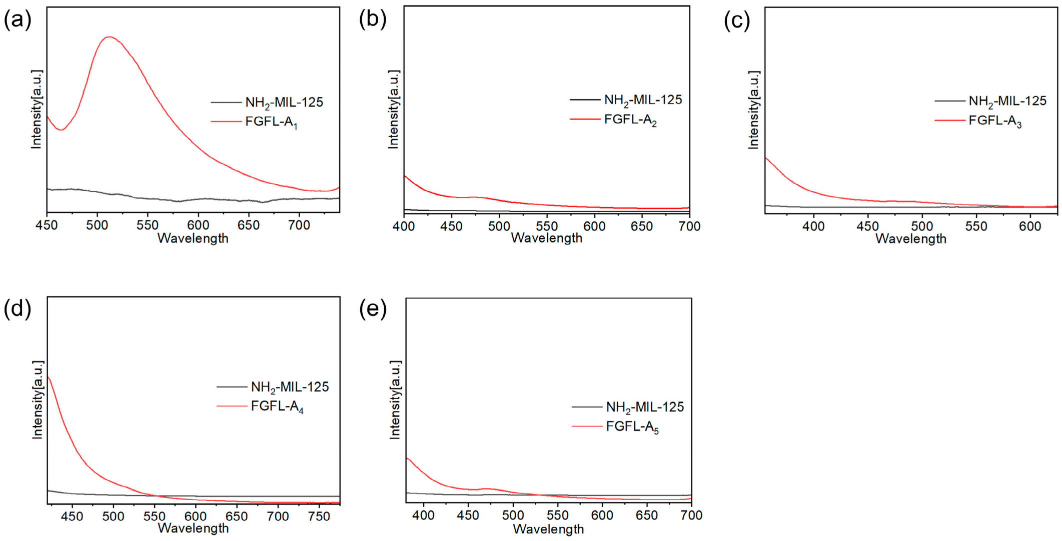

Figure S3. Fluorescence spectra of (a) FGFL-A_1, (b) FGFL-A_2, (c) FGFL-A_3, (d) FGFL-A_4 and (e) FGFL-A_5.

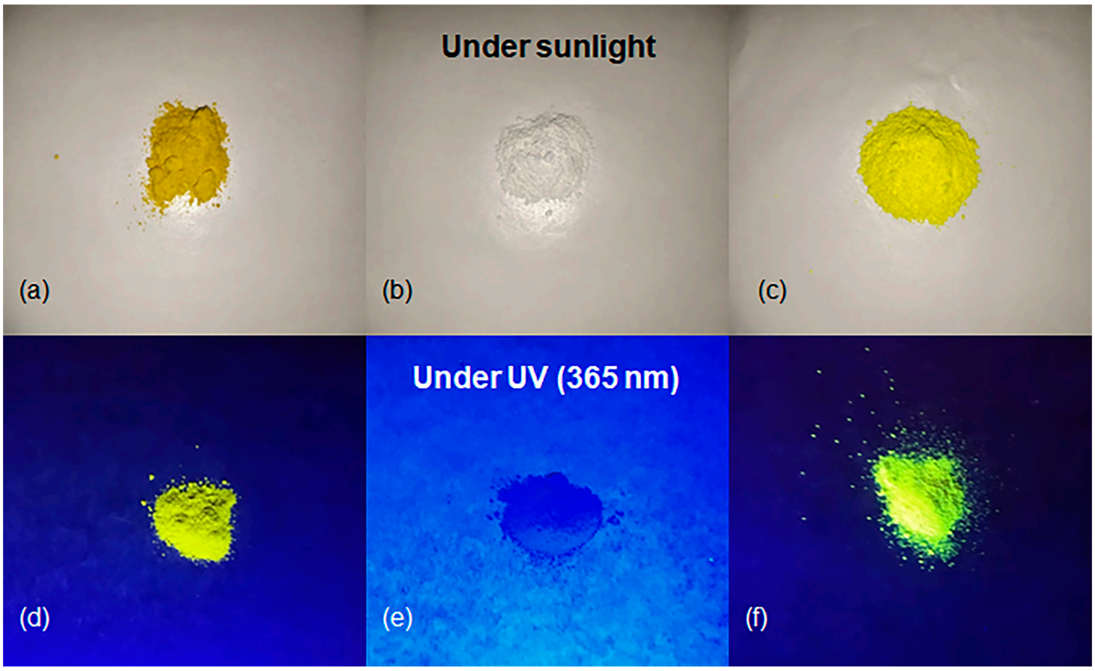

Figure S4. Electronic photos of ThT, MIL-125, and FGFL-B_1 (a-c) under sunlight and (d-f) under UV (365 nm).

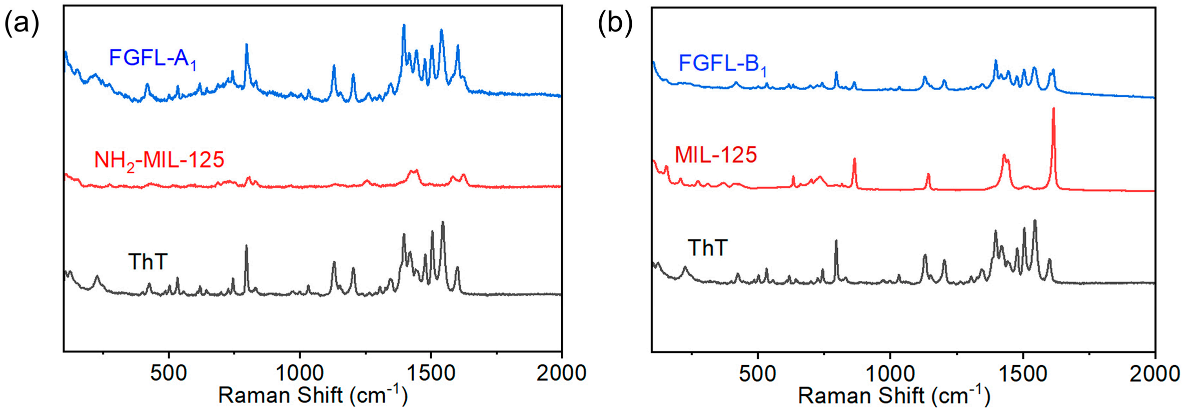

Figure S5. Raman spectra of (a) ThT, NH_2-MIL-125 and FGFL-A_1, (b) ThT, MIL-125 and FGFL-B_1.

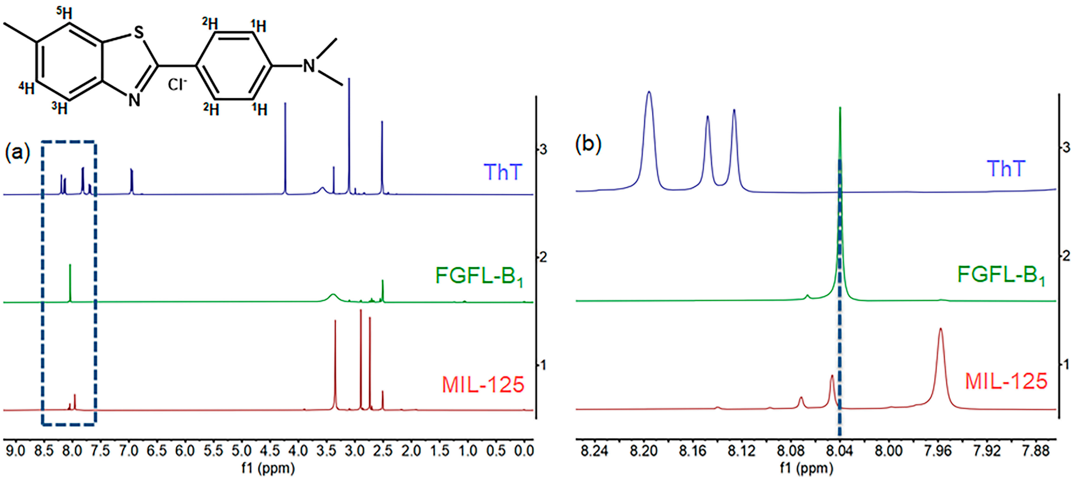

Figure S6. (a) The liquid-state ^1H NMR spectra in dimethyl sulfoxide of ThT, FGFL-B_1 and MIL-125; (b) The liquid-state ^1H NMR spectra in dimethyl sulfoxide of ThT, FGFL-B_1, MIL-125(7.87-8.25 ppm).

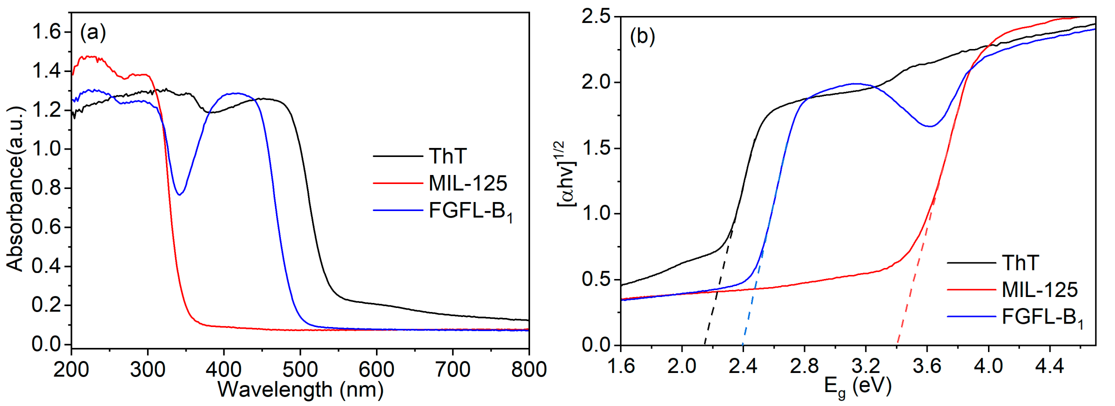

Figure S7. (a) The UV-vis absorption spectra of ThT, MIL-125 and FGFL-B_1; (b) The plot of $(ahv)^{1/2}$ versus hv of ThT, MIL-125, and FGFL-B_1

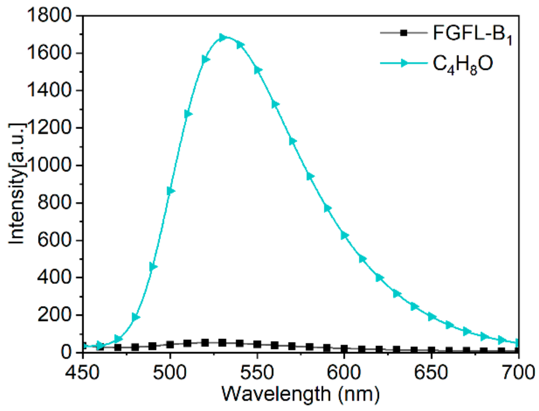

Figure S8. The fluorescence spectra of FGFL-B_1 and FGFL-B_1 loaded with THF.

Table S2. Adsorption properties of FGFL-B_1 for the 6 selected VOCs.

Samples	Adsorption property/mg g^{-1}
C_6H_6	378
$C_6H_5CH_3$	200
CCl_4	812
CH_2Cl_2	1230
HCHO	80
C_4H_8O	655

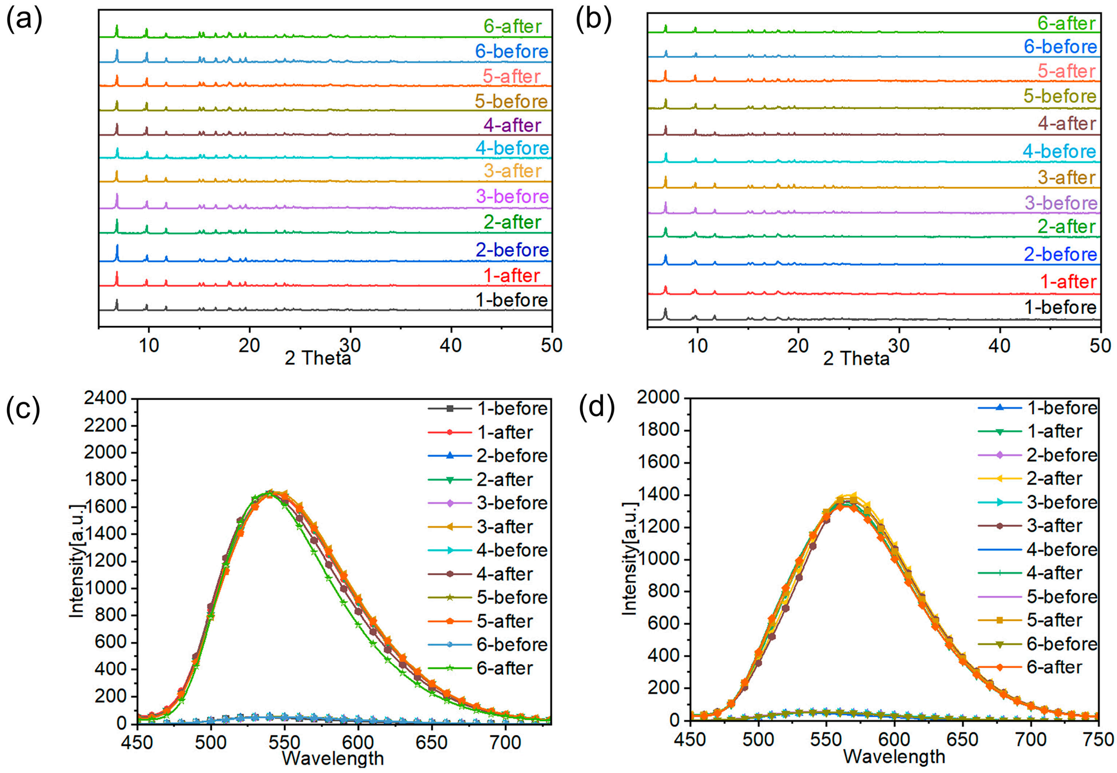

Figure S9. After FGFL-B_1 adsorbed VOCs, the VOCs were removed by vacuum drying for 333 K. XRD patterns and fluorescence intensity were measured before and after the cycle stability experiment. The numbers 1, 2, 3, 4, 5, 6 represent the number of adsorption and desorption cycles. A general survey of the PXRD patterns of (a) FGFL-B_1 before and after loaded with CCl_4, (b) FGFL-B_1 before and after loaded with THF. A general survey of the fluorescence spectra of (c) FGFL-B_1 before and after loaded with CCl_4, (d) FGFL-B_1 before and after loaded with THF.

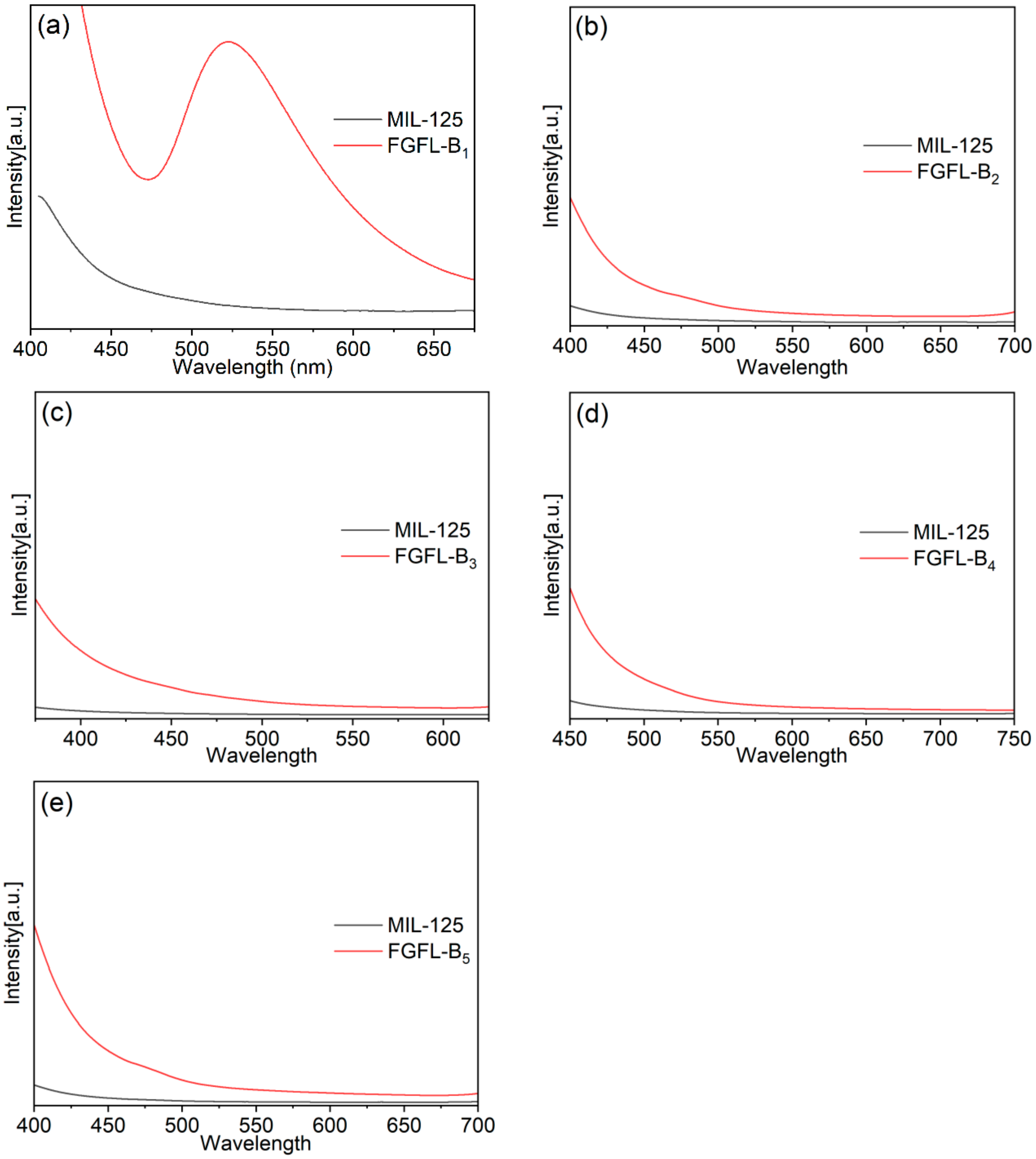

Figure S10. Fluorescence spectra of (a) FGFL-B_1, (b) FGFL-B_2, (c) FGFL-B_3, (d) FGFL-B_4 and (e) FGFL-B_5.
